# Non-coding RNAs in Cardiac Regeneration

**DOI:** 10.3389/fphys.2021.650566

**Published:** 2021-03-24

**Authors:** Ting Yuan, Jaya Krishnan

**Affiliations:** Institute of Cardiovascular Regeneration, Center for Molecular Medicine, Goethe University Frankfurt, Frankfurt am Main, Germany

**Keywords:** ncRNA, miRNA, lncRNA, cardiac regeneration, cardiomyocyte proliferation, cardiac differentiation, cardiac reprogramming, cardiomyocyte apoptosis

## Abstract

The adult heart has a limited capacity to replace or regenerate damaged cardiac tissue following severe myocardial injury. Thus, therapies facilitating the induction of cardiac regeneration holds great promise for the treatment of end-stage heart failure, and for pathologies invoking severe cardiac dysfunction as a result of cardiomyocyte death. Recently, a number of studies have demonstrated that cardiac regeneration can be achieved through modulation and/or reprogramming of cardiomyocyte proliferation, differentiation, and survival signaling. Non-coding RNAs (ncRNAs), including microRNAs (miRNAs), long non-coding RNAs (lncRNAs) and circular RNAs (circRNAs), are reported to play critical roles in regulating key aspects of cardiomyocyte physiologic and pathologic signaling, including the regulation of cardiac regeneration both *in vitro* and *in vivo*. In this review, we will explore and detail the current understanding of ncRNA function in cardiac regeneration, and highlight established and novel strategies for the treatment of heart failure through modulation of ncRNAs-driven cardiac regeneration.

## Introduction

Despite improvements in interventional and pharmacological therapies, cardiovascular disease is still the leading cause of hospitalization and mortality in the industrial world. Heart failure, in particular, affects 64 million people worldwide and is a major cause of cardiovascular morbidity and mortality with tremendous impact on healthcare systems and economic productivity (Disease et al., 2017). Heart failure is classified into three primary indication groups depending on the left ventricular ejection fraction (EF): heart failure with reduced ejection fraction (HFrEF), heart failure with preserved ejection fraction (HFpEF), and heart failure with mid-range ejection fraction (HFmEF) (Ponikowski et al., 2016). Loss of cardiomyocytes is a key hallmark of heart failure, in both age- and myocardial infarction (MI)-related heart failure. During MI, around 25% of cardiomyocytes die and are replaced by fibrotic scar tissue (Murry et al., 2006). However, the remaining cardiomyocytes have very limited regenerative and repair capacity to restore the damaged tissue. Thus, these changes lead to cardiac remodeling and fibrosis, culminating in heart failure and mortality (He and Zhou, 2017). Currently, heart transplantation is the primary treatment of choice for end-stage heart failure as a means to renew impaired heart function. However, heart transplantation is restricted by the limited availability of donor organs, treatment costs and surgical complexities. Thus, the unmet need for new therapies in the treatment of heart failure remains very high, and cardiac regenerative strategies targeting the replenishment and replacements of lost cardiomyocytes hold great promise and potential.

Non-coding RNAs (ncRNAs) are functional transcripts that are mainly transcribed by RNA polymerase II and share several characteristics with coding messenger RNAs (mRNAs). ncRNAs can be divided into two main groups: the small ncRNAs (< 200 nt) and the long ncRNAs (lncRNAs, > 200 nt) (Garreta et al., 2017). Among the various types of ncRNAs, microRNA (miRNAs), lncRNAs and circular RNAs (circRNAs) are the three major classes of ncRNAs and have attracted increasing attention. Recently, many studies demonstrate that a large number of ncRNAs, including miRNAs, lncRNAs and circRNAs, have been identified to regulate genetic networks through modulation of transcriptional modulation and epigenetic, and sequentially govern cardiomyocyte fate and potentially mediate cardiac regeneration (Hobuss et al., 2019; Abbas et al., 2020; Braga et al., 2020). Gain- and loss-of-function approaches demonstrate a key role of ncRNAs in regulating cardiac regeneration in both *in vitro* and *in vivo* models.

In this review, we will focus on the current understanding of the roles of ncRNAs in the process of cardiac regeneration, including cardiomyocyte proliferation, cardiomyocyte survival, cardiac differentiation and cardiac reprogramming. Based on this scientific knowledge we will also discuss the strategy of targeting ncRNAs for cardiac regeneration therapy.

## Part 1: Cardiac Regeneration

The mammalian heart cell is considered as being post-mitotic due to its negligible proliferative capacity (Doppler et al., 2017). It was previously thought that the total number of cardiomyocytes is established after birth and cardiomyocytes of adult mammals are permanently quiescent. However, an ever increasing body of evidence suggests that turnover of cardiomyocytes does in fact occur in the adult mammalian heart. It has been show that the hearts of 1-day-old neonatal mice are able to regenerate following partial cardiac resection, but this capacity is lost by 7 days of age (Porrello et al., 2011b). Similarly, neonatal human hearts have the regeneration capacity to repair myocardial damage after MI and completely recover cardiac function (Haubner et al., 2016). Furthermore, a study by Bergmann et al. (2009, 2015) revealed that cardiomyocyte annual turnover gradually decreases from a rate of 1% at the age of 20, to 0.3% at the age of 75, while other cell types, including endothelial and mesenchymal cells, maintain annual turnover rates of around 20%. This suggests that adult cardiomyocytes have very limited proliferative and regenerative capacity, and are thus incapable of replacing damage cardiomyocytes to restore normal cardiac function after myocardial injury. Thus, an urgent need has emerged for new therapeutic strategies to enhance the regenerative capacity of cardiomyocytes to restore the functional capacity of the diseased myocardium.

Despite tremendous advances in cardiac repair and cardiac regenerative medicine, current therapies for cardiac regeneration following myocardial infarction are limited and non-curative. Over the past several decades, cardiac regeneration has been the central target for restoring the injured heart and has been shown to be achievable through several approaches: (1) cell transplantation strategy, (2) direct or indirect reprogramming non-myocytes into cardiomyocyte-like cells, (3) enhance the proliferation of endogenous cardiomyocytes, (4) decrease cardiomyocyte apoptosis (Choong et al., 2017). For strategy 1, multiple studies have used induced pluripotent stem cells (iPSCs) and embryonic stem cells (ESCs) to generate new cardiomyocytes *ex vivo* for transcoronary delivery or transplantation into infarcted or injured regions of the heart (Xin et al., 2013b). For strategy 2, reprogramming of cardiac fibroblasts to the cardiomyocyte lineage has been proposed as an alternative cell-free approach for cardiac regeneration (Song et al., 2012). For strategies 3 and 4, enhancing proliferation of endogenous cardiomyocytes or improving cardiomyocyte survival in the face of pathologic stress has been proposed (Xin et al., 2013a; Hashimoto et al., 2018). In this review, we will elucidate a number of ncRNAs implicated in four aspects of cardiac regeneration: cardiomyocyte proliferation, differentiation, reprogramming and survival.

## Part 2: miRNAs and Cardiac Regeneration

### miRNA Biology

MicroRNAs (miRNAs) are small, ∼ 22 nucleotide non-coding RNAs that control patterns of gene expression by binding to 3′-untranslated region (3′-UTR) of their targeted mRNAs thereby promoting mRNA degradation or inhibiting mRNA translation (Filipowicz et al., 2008). miRNAs have been demonstrated to play essential roles in proliferation, apoptosis, differentiation and development of many cells and tissues including the heart (Porrello, 2013; Hodgkinson et al., 2015).

MicroRNAs biogenesis is initiated by generation of primary miRNA (pri-miRNA) transcripts. The pri-miRNA are transcribed from DNA sequences by RNA polymerase II and then processed into precursor miRNAs (pre-miRNAs) by the microprocessor complex, consisting of RNase III enzyme Drosha and dsRNA-binding protein DiGeorge Syndrome Critical Region 8 (DGCR8) (Denli et al., 2004). Thereafter, the pre-miRNAs are transported into the cytoplasm via the nuclear export factor exportin 5 (EXP-5)/RanGTP complex and then processed by the RNase III endonuclease Dicer to produce ∼ 22 nucleotide mature miRNA duplexes (Zhang et al., 2004). The mature miRNA duplexes can be loaded into the Argonaute (AGO) family of proteins (AGO 1-4 in humans) in an ATP-dependent manner, and then unwound into the single miRNA guide strand by the RNA-induced silencing complex (miRISC). Then the miRISC complexes specifically bind to the 3′-UTR of target mRNAs and inhibits their translation or leads to their degradation (Bartel, 2004).

**FIGURE 1:**
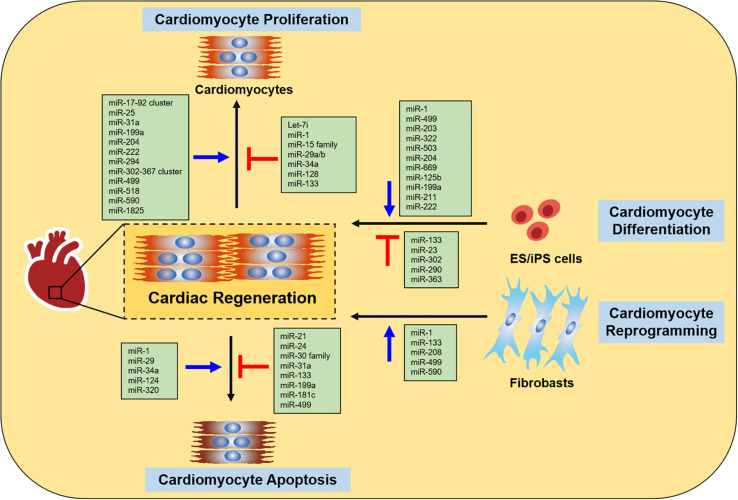
miRNAs in cardiac regeneration. The figure summarizes the function of miRNAs in regulating cardiac regeneration, including cardiomyocyte proliferation, cardiomyocyte differentiation, cardiomyocyte reprogramming and cardiomyocyte apoptosis.

Currently, many studies indicate that miRNAs are involved in various specialized biological processes during cardiac development, disease and ultimately cardiac regeneration and repair (Porrello, 2013; Wu et al., 2013; Hodgkinson et al., 2015). A large number of miRNAs have been shown to participate in regulating cardiac regeneration through controlling cardiomyocyte proliferation, cardiomyocyte differentiation, cardiomyocyte reprogramming, and cardiomyocyte survival *in vitro* and *in vivo* (Figure 1).

### miRNAs and Cardiomyocyte Proliferation

The proliferation capacity of mammalian cardiomyocyte is robust during the fetal period but is switched off early after birth (Gunthel et al., 2018). A number of miRNAs have been identified that induce or inhibit cardiomyocyte proliferation *in vitro* and *in vivo*. A high-throughput functional phenotypic screen using a whole-genome miRNA libraryidentifies 204 potential human miRNAs that are able to promote cardiomyocyte proliferation in neonatal rat cardiomyocytes as determined by 5-ethynyl-2′-deoxyuridine (EdU)-incorporation (Eulalio et al., 2012). Among the 204 miRNAs identified, miR-199a, miR-302b, miR-518, miR-590 and miR-1825 are shown to increase EdU-incorporation, Ki-67, and phospho-histone H3 (pHH3) in mouse or rat cardiomyocytes (Eulalio et al., 2012). Moreover, miR-199a and miR-590 were linked to activation of cell cycle induction and progression, and the enhancement of cardiomyocyte proliferation *in vitro* and *in vivo* (Eulalio et al., 2012). Furthermore, the induction of cardiomyocyte proliferation induced by these two miRNAs led to improved cardiac function and decrease cardiac fibrosis in response to myocardial infarction (MI) (Eulalio et al., 2012). An independent proliferation screen performed in human induced pluripotent stem cell-derived cardiomyocytes (hiPSC-CMs) identified 96 miRNAs as drivers of DNA synthesis and cell division in cardiomyocytes (Diez-Cunado et al., 2018). Additional chemical screening and computational approaches revealed that 67 of 96 miRNAs stimulated cardiomyocyte proliferation in a yes-associated protein (YAP)-dependent manner in hiPSC-CMs (Diez-Cunado et al., 2018).

Other studies have implicated additional miRNAs in cardiomyocyte proliferative induction, including the miR-17-92 cluster, miR-25, miR-31a, miR-204, miR-222, miR-294, miR-302-367 cluster, miR-499 and miR-1825. The miR-17-92 cluster, one of the best-characterized miRNA clusters, encodes six mature miRNA members including miR-17, miR-18a, miR-19a, miR-19b, miR-20a and miR-92a (Mogilyansky and Rigoutsos, 2013). A recently study has shown that overexpression of the miR-17-92 cluster induces cardiomyocyte proliferation in embryonic, postnatal as well as in adult heart and rescues cardiac function after MI by inhibiting phosphatase and tensin homolog (PTEN) (Chen et al., 2013; Shi et al., 2017). In contrast, inhibition of miR-17 *in vivo* was shown to attenuate excise-induced cardiac growth and cardiomyocyte proliferation (Shi et al., 2017). The combination of miR-19a and miR-19b led to increased cardiomyocyte proliferation and protected heart against MI in mice (Gao et al., 2019). miR-25, which has the same seed sequences as miR-92a, is also able to stimulate cardiomyocyte proliferation in neonatal rat cardiomyocytes (NRCs) by targeting the Bcl-like protein BCL2 like 11 (Bim) (Qin et al., 2019). Another study has also shown that miR-25 overexpression promotes cardiomyocyte proliferation in hPSC-CMs by targeting F-Box And WD Repeat Domain Containing 7 (FBXW7) (Wang et al., 2020). Overexpressing miR-204 improves cardiomyocyte proliferation via targeting Jarid2, resulted in the upregulation of cell cycle regulator Cyclin A, Cyclin B, cyclin D2, Cyclin E, CDC2 and PCNA *in vitro* and *in vivo* (Liang et al., 2015). miR-31a, is significantly upregulated in post-natal day 10 (P10) cardiomyocytes compared to P0, has also been shown to regulate cardiomyocyte cell cycle progression (Xiao et al., 2017). Overexpression of miR-31a in neonatal rat ventricular myocytes (NRVM) and in rat neonates induces cardiomyocyte proliferation by targeting Rho Related BTB Domain Containing 1 (RhoBTB1) (Xiao et al., 2017). In addition, overexpression of miR-222 in mice increased cardiomyocyte proliferation after ischemia/reperfusion (I/R) injury, whereas miR-222 inhibition reduced cardiomyocyte proliferation in response to physical exercise (Liu et al., 2015; Vujic et al., 2018). miR-302-367 cluster is expressed in the embryonic mouse heart and lost in the adult heart, has also been shown to regulate cardiomyocyte proliferation. Loss of miR-302-367 cluster led to decreased cardiomyocyte proliferation in mice, while overexpression of miR-302-367 cluster improved cardiomyocyte proliferation and decreased cardiac fibrosis after MI through repression of the Hippo pathway (Tian et al., 2015). miR-410 and miR-495 both belong to Gtl2-Dio3 miRNAs and have been reported to promote cardiomyocyte proliferation through suppression of CREB binding protein (CBP)/p300 interacting transactivator with Glu/Asp rich carboxy-terminal domain 2 (Cited2) as determined by increased EdU incorporation and Ki67 in neonatal cardiomyocytes (Clark and Naya, 2015). miR-294, which is expressed in the heart during development, increased cardiomyocyte proliferation, improved cardiac function and decreased infarct size after MI via suppression of Wee1 *in vivo* (Borden et al., 2019). miR-499, a myocyte-specific miRNA (myomiR) is expressed within the one of the introns of β-myosin heavy chain (Myh7b) gene, could also promote neonatal cardiomyocyte proliferation by regulating SRY-Box Transcription Factor 6 (Sox6) and cyclin D1 (Li et al., 2013). A recent study demonstrated that miR-1825 increased both DNA synthesis and cytokinesis in adult cardiomyocytes and improve cardiac function after MI (Pandey et al., 2017).

In addition to miRNAs that promote cardiomyocyte proliferation, several miRNAs that are endogenously expressed in cardiomyocytes have been shown to suppress cardiomyocyte proliferation, including let-7i, miR-1, miR-15 family, miR-29a/b, miR-34a, miR-128, miR-128, miR-133 (Braga et al., 2020). Let-7 was one of the first miRNAs isolated and characterized in the nematode *C. elegans*. Overexpression of let-7i inhibits cardiomyocyte proliferation, whereas the suppression of let-7i induces cardiomyocyte proliferation and improves cardiac function in response to MI by enhancing E2F Transcription Factor 2 (E2F2) and Cyclin D2 (Hu et al., 2019). miR-1 and miR-133 are highly conserved and expressed in cardiac and skeletal muscle and are transcriptionally regulated by myogenic transcription factors MyoD, myocyte enhancer factor-2 (Mef2) and serum response factor (SRF) (Zhao et al., 2007). Overexpression of miR-1 in the developing hearts leads to decreased cardiomyocyte proliferation by targeting the heart and neural crest derivatives-expressed protein 2 (Hand 2) transcription factor (Zhao et al., 2005). Conversely, miR-1 deletion increases cardiomyocyte proliferation in the adult hearts (Zhao et al., 2007). Similar to miR-1, overexpression of miR-133a restricts cardiomyocyte proliferation in zebrafish (Yin et al., 2012), while miR-133a inhibition increases cardiomyocyte proliferation in adult hearts (Liu et al., 2008). The miR-15 family (including miR-15a, miR-15b, miR-16-1, miR-16-2, miR-195 and miR-497), modulates neonatal heart regeneration through inhibition of postnatal cardiomyocyte proliferation and repression of cell cycle genes, thus resulting in postnatal loss of cardiac regenerative capacity (Porrello et al., 2013). Inhibition of miR-15 family from the early postnatal period until adulthood promotes cardiomyocyte proliferation and enhances cardiac function in response to MI (Hullinger et al., 2012; Porrello et al., 2013). Overexpression of miR-195 in the embryonic heart suppresses cardiomyocyte proliferation and impairs the regeneration capability of 1-day-old mouse heart after MI through the repression of a number of cell cycle genes, including checkpoint kinase 1 (Chek1) *in vivo* (Porrello et al., 2011a, 2013). Inhibition of miR-26a increased proliferation of mouse neonatal cardiomyocytes *in vitro* and *in vivo* via regulation of cell cycle inhibitors (Crippa et al., 2016). Overexpression of miR-29a suppresses cardiomyocyte proliferation, while its inhibition enhances cell division by inducing cyclin D2 expression in NRCs (Cao et al., 2013). Similar to miR-29a, inhibition of miR-29b promotes cardiomyocyte proliferation by inactivation of notch receptor 2 (NOTCH2) function *in vitro* and *in vivo* (Yang et al., 2020). miR-34a, a regulator of age-associated physiology, also suppresses cardiomyocyte proliferation and its inhibition leads to enhanced cardiomyocyte proliferation and improves cardiac function in response to MI through targeting of silent information regulator factor 2 related enzyme 1 (Sirt1), B-cell lymphoma 2 (Bcl2) and Cyclin D1 (Yang et al., 2015). Inhibition of miR-128 promotes cardiomyocyte proliferation by activating cyclin E- and cyclin dependent kinase 2 (CDK2)-positive cell cycle regulators and improves cardiac function in response to MI (Huang et al., 2018).

### miRNAs and Cardiac Differentiation

In addition to enhancing the proliferative capacity of endogenous cardiomyocytes, transplanting cardiac stem cells or cardiomyocytes derived from iPSCs or ESCs is another therapeutic approach in the field of cardiac regeneration and is considered a promising strategy to heart failure. Pluripotent stem cells provide great promise for regenerative medicine due to their self-renewal potential and ability to differentiate into multiple cell lineages or specific functional cell types. Several studies have shown that miRNAs play a key role in differentiation of stem cells, including miR-1, miR-23, miR-125b, miR-133, miR-199a, miR-203, miR-204, miR-211, miR-222, miR-290, miR-302, miR-499, miR-669.

miR-1 and miR-499 are also highly enriched in cardiomyocytes and upregulated in differentiated cells. Studies have shown that miR-1 promotes cardiac differentiation from ESCs by the downregulation of Notch ligand Delta-like 1 (Chen et al., 2006). Moreover, overexpression of miR-1 itself is sufficient to promote cardiac differentiation mediated by upregulation of the cardiac differentiation genes nk2 homeobox 5 (NKX2.5) and myosin heavy chain beta (β-MHC) in mouse and human ESCs (Ivey et al., 2008). In addition, Sluijter and colleagues have also shown that miR-1 overexpression enhances human cardiomyocyte progenitor cells (hCMPCs) differentiation into cardiomyocytes by repression of Histone Deacetylase 4 (HDAC4) (Sluijter et al., 2010). Furthermore, transplantation of mouse ESCs overexpressing miR-1 (miR-1-ES cells) into the border zone of the infarcted hearts significantly promotes cardiac differentiation and protects against MI-induced apoptosis through activation of PI3K/Akt pathway (Glass and Singla, 2011). Similarly, miR-499 overexpression enhances differentiation of cardiac progenitors into cardiomyocytes in human CMPCs and ESCs by inhibition of Sox6 in human CMPCs (Sluijter et al., 2010). In addition, overexpression of miR-499 significantly enhances cardiac differentiation in human ESC-derived cardiomyocytes by upregulation of cardiac myosin heavy chain genes and cardiac transcription factor MEF2C (Wilson et al., 2010).

In addition to miR-1 and miR-499, overexpression of miR-203 improves efficient differentiation and maturation to cardiomyocytes by repression of DNA methyltransferase 3a/b (Dnmt3a/b) in mouse iPSCs (Salazar-Roa et al., 2020). miR-322 and miR-503 clusters have shown that participate in the specification into cardiomyocyte progenitors of the mesodermal cells by the repression of CUGBP Elav-like family member (Celf) family proteins (Shen et al., 2016). miR-204, miR-669 and miR-23 have also been shown to promote cardiac progenitor differentiation (Crippa et al., 2011; Xiao et al., 2012). Furthermore, recently another study shows that a combination of miRNAs (miR-125b, miR-199a, miR-221 and miR-222) promotes the maturation of both mouse and human cardiomyocytes differentiated from ESCs (Lee et al., 2015).

miR-1 and miR-133 are co-transcribed due to their polycistronic clustering on the same chromosome. However, they have antagonistic effects on lineage commitment and terminal differentiation, as miR-133 inhibits cardiomyocyte terminal differentiation and maintains them in a proliferative state (Chen et al., 2006). Overexpression of miR-133 suppresses the expression of cardiac markers in human and mouse ESCs, and inhibits differentiation of ESCs into CMs (Alfar et al., 2018), while miR-133 deletion promotes cardiomyocyte proliferation partly via activation of SRF and G1/S-specific cyclin D2 (Chen et al., 2006; Liu et al., 2008), thus highlighting the inhibitory role of miR-133 in both cardiomyocyte differentiation and proliferation. Recently, a study revealed that inhibition of miR-23 facilitated the transformation of bone marrow mesenchymal stem cells (BMSCs) into myocardial cells and conferred protection against Ischemia/reperfusion (I/R)-induced myocardial damage by activation of Wingless and Int-1 (Wnt) pathway (Lu et al., 2019). miR-302/miR-290 members are involved in the maintenance of pluripotency of murine ES cells, and the members of these families have been shown to suppress cell differentiation (Judson et al., 2009; Melton et al., 2010). In addition, miR-363 negatively regulates in cardiomyocyte specification, miR-363 inhibition leads to an enrichment of left ventricular ESC-derived cardiomyocytes by targeting Hand 1 (Wagh et al., 2014).

Taken together, these studies demonstrate that miRNAs regulate cardiomyocyte differentiation and help balance cardiomyocyte differentiation with proliferation, which could be further translated clinically by using miRNAs to induce transferred ESCs/iPSCs to differentiate into mature cardiomyocytes to replace damaged cardiomyocytes.

### miRNAs and Cardiac Reprogramming

There are two methods for cardiomyocyte reprogramming: indirect reprogramming and direct reprogramming. The indirect reprogramming uses an intermediary step where the non-myocytes dedifferentiate into progenitor cells that then could be further programmed into the cardiomyocytes (i.e., iPSC reprogramming). The direct reprogramming directly converts the non-myocytes into cardiomyocytes without passing through the pluripotent state (Farber and Qian, 2020). Over the last decade, direct reprogramming from cardiac fibroblasts of the infarcted area into cardiomyocytes has been successful in the repair of damaged heart tissue, and has great promise for the clinic.

miRNAs are able to directly induce the cellular reprogramming of cardiac fibroblasts into cardiomyocytes *in vitro* and *in vivo* (Rao et al., 2006; Jayawardena et al., 2012; Joladarashi et al., 2014). Recently, Jayawardena et al. has identified and evaluated a specific combination of miRNAs (miR-1, miR-133, miR-208 and miR-499; miR combo) which is able to directly reprogram cardiac fibroblasts into cardiomyocyte-like cells *in vitro* and *in vivo* (Jayawardena et al., 2012). *In vitro*, the reprogrammed cardiomyocyte-like cells express cardiomyocyte-specific genes, and exhibit sarcomeric organization and functional properties characteristic of mature cardiomyocytes (Jayawardena et al., 2012). Furthermore, injection of lentivirus miR combo into the border zone of the infarcted heart also induced generation of new cardiomyocyte-like cells from lineage-traced non-cardiac myocytes by 4 weeks post myocardial injury (Jayawardena et al., 2012). In addition, miR-combo significantly decreases cardiac fibrosis and improves cardiac function, as indicated by the improvement of fractional shortening (FS) and ejection fraction (EF) following MI (Jayawardena et al., 2015). These data demonstrate that administration of miRNAs to the peri-infarct area of the infarcted heart *in vivo* is able to direct reprogramming of cardiac fibroblasts into functional cardiomyocytes.

Recent studies demonstrate that co-overexpression of the GATA binding protein 4 (Gata4), Mef2c, and T-box transcription factor 5 (Tbx5), termed GMT, leads to the reprogramming of cardiac fibroblasts into cardiomyocytes *in vitro* and *in vivo* (Ieda et al., 2010; Qian et al., 2012). Additionally, they observe that the combination of miR-133 and GMT further enhanced cardiac reprogramming of mouse embryonic fibroblasts into cardiomyocytes in terms of kinetics and efficiency by directly binding to the snail family transcriptional repressor 1 (Snail 1) (Jayawardena et al., 2012). Interestingly, a study by Singh and colleagues reveal that miR-590, which is a key miRNA in cardiomyocyte proliferation, could also further enhance GMT-mediated reprogramming in human and pig fibroblasts by suppressing the Sp1 transcription factor (Sp1), that can inhibit the activity of cardiac-specific genes (Singh et al., 2016). In addition, another study shows that the combination of miR-1, miR-133 and Gata4, Mef2c, TBx5, Myocardin could further increase the direct reprogramming efficiency of human fibroblasts to cardiomyocyte-like cells (Nam et al., 2013).

### miRNAs and Cardiomyocyte Survival

Cardiomyocyte survival is recognized as being a central feature of cardiac remodeling and progression in heart failure (Briasoulis et al., 2016). The underlying triggers of cell survival and development of novel treatment strategies to suppress cardiomyocyte apoptosis is important. Cardiomyocyte apoptosis can be triggered by the activation of two major signaling pathways: the intrinsic and extrinsic pathways. The intrinsic pathway is mediated by mitochondrial membrane permeabilization and the extrinsic pathway involving the activation of surface death receptor by death ligands (Bennett, 2002). Several miRNAs have been shown to pro-apoptotic and anti-apoptotic regulate apoptotic signaling pathways, which may have to effect on survival of cardiomyocytes. miR-21, miR-24, miR-30 family, miR-31a, miR-133, miR-138, miR-199a, miR-181c, and miR-499 are main miRNAs that could inhibit cardiomyocyte apoptosis. The miR-1, miR-29, miR-34a, miR-124, and miR-320 exhibit an pro-apoptotic effect in cardiomyocytes.

Many studies demonstrate that miR-21 promotes cardiac survival and attenuates cardiomyocyte apoptosis *in vitro* and *in vivo*. Overexpression of miR-21 protects against hydrogen peroxide (H_2_O_2_)-induced cardiac apoptosis via inhibition of programmed cell death protein 4 (pDCD4) and activator protein 1 (AP-1) pathway *in vitro* (Cheng et al., 2009). miR-21 is decreased in the infarct zone after MI, while miR-21 overexpression in rat hearts reduced cardiac fibrosis and decreased cardiomyocyte apoptosis in the infarct and border zone after AMI (Dong et al., 2009; Yan et al., 2015). Furthermore, overexpression of miR-21 alone significantly inhibits autophagic activity and alleviates hypoxic/reoxygenation (H/R)-induced cardiac apoptosis by regulation of PTEN/Akt/mTOR pathway (Huang et al., 2017). In addition, combination of miR-21 and miR-146 could further decrease ischemic/Hypoxic-induced cardiomyocyte apoptosis compared to either miR-21 or miR-146a alone in NRCs (Huang et al., 2016). Furthermore, simultaneous delivery of agomiR-21 and agomiR-146a in mice attenuates cardiomyocyte apoptosis and improves cardiac function following AMI by regulation of p38 MAPK (Huang et al., 2016). *In vivo* studies show that overexpression of miR-24 protects against cardiomyocyte apoptosis and mediated by repression of BH3-domain-caontaining protein (Bim) in response to MI in mice (Qian et al., 2011). miR-30 family members, including miR-30a, miR-30b and miR-30d, inhibits mitochondrial fission through suppressing the expression of p53 and downstream targets dynamin-related protein-1 (Drp1), leading to decreased cardiomyocytes apoptosis (Li et al., 2010). Furthermore, doxorubicin-induced apoptosis could be rescued by miR-30 expression in rat cardiomyocytes *in vivo* (Roca-Alonso et al., 2015). miR-31a is upregulated in the failing heart, overexpression of miR-31a protects against the angiotensin II (AngII)-induced apoptosis and caspase-3 activity by targeting Tp53 in the cardiac H9C2 cell line (Yan et al., 2018). Gain-of -function studies demonstrate that miR-133 overexpression inhibits cardiac apoptosis and enhances cardiac function following chronic pressure overload. miR-133 overexpression decreases I/R-induced apoptosis, whereas miR-133 deletion increases cardiac apoptosis mediated by regulating the expression of casp9 (He et al., 2011). miR-199a also plays a crucial role in hypoxia-induced apoptosis (Rane et al., 2009). Replenishing miR-199a during hypoxia decreases cardiomyocytes apoptosis through inhibition of hypoxia-inducible factor (Hif)-1α and its stabilization of p53, while miR-199a inhibition recapitulates hypoxia preconditioning by up-regulating Hif-1α and Sirt1 (Rane et al., 2009). miR-181c is suppressed in the heart tissue of doxorubicin (DOX)-induced heart failure animal model (Li et al., 2020). Study shows that miR-181c overexpression protects heart failure by impeding cardiomyocyte apoptosis through PI3K/Akt pathway (Li et al., 2020). Overexpression or knockdown of miR-499 decreases or increases the cardiomyocyte apoptosis *in vitro* (Li et al., 2016). In addition, overexpression of miR-499 decreases cardiac apoptosis and reduces myocardial infarct size in the rat AMI models by inhibiting PDCD4 (Li et al., 2016).

In addition to miRNAs that reduce cardiomyocyte apoptosis, there are several miRNAs that exert the opposite effect on cardiomyocyte apoptosis. miR-1, which has been shown to play an essential role in the regulation of cardiac proliferation and differentiation, also plays a critical role in cardiomyocyte apoptosis. Overexpression of miR-1 in mice increases cardiomyocyte apoptosis, while deletion of miR-1 reduces I/R-induced cardiomyocyte apoptosis and attenuates cardiac I/R injury by targeting protein kinase C epsilon (PKCε) (Pan et al., 2012). Overexpression of miR-29 promotes apoptosis, whereas inhibition of miR-29 reduces cardiomyocyte apoptosis and infarct size in hearts subjected to I/R injury by decreasing the expression of pro-apoptotic molecular Bcl-2-associated X protein (Bax) and increasing anti-apoptotic molecular Bcl2 (Ye et al., 2010). miR-34a is induced in the heart during aging in both mice and humans, and its deletion *in vivo* reduces age-induced cardiomyocyte cell death and fibrosis, and improves cardiac function after AMI by activation of serine/threonine-protein phosphatase 1 regulatory subunit 10 (PNUTS) (Boon et al., 2013). miR-124 is upregulated in a mice model of MI, inhibition of miR-124 decreases MI-induced cardiomyocyte apoptosis and infarct size and improves cardiac function in mice by upregulating signal transducer and activator of transcription 3 (STAT3) (He et al., 2018). miR-320 is significantly decreased in the hearts with I/R injury *in vivo* and *ex vivo*, and its overexpression enhances cardiomyocyte apoptosis and death in the hearts on I/R (Ren et al., 2009). Conversely, administration of antagomir-320 reduces I/R-induced cardiac injury and cardiomyocyte apoptosis by targeting heat-shock protein 20 (Hsp20) (Ren et al., 2009).

Together, these studies indicate that miRNAs can effectively stimulate cardiac regeneration by directly modulating target gene expression, and provide promising a therapeutic avenue for heart failure treatment. To date, a large number of miRNAs have been discovered to regulate cardiac regeneration. However, the molecular targets and mechanisms underlying miRNA function needs to be further investigated, and the miRNA-based preclinical and clinical trials need to be better and more stringently assessed for therapeutic efficacy.

## Part 3: LncRNAs and Cardiac Regeneration

Long non-coding RNAs (LncRNAs) are defined as RNA transcripts over 200 nucleotides in length with no evidence for a protein-coding function (Mercer et al., 2009). There are our major classes of lncRNAs: Antisense lncRNAs, Bidirectional lncRNAs, Intergenic lncRNAs and Sense-intronic lncRNAs. The role of lncRNAs in controlling cardiac regeneration has only been studied in recent years.

Similarly to miRNAs, lncRNAs have been shown to regulate cardiomyocyte proliferation either by enhancing or suppressing cell cycle progression (Table 1). The Sirt1 antisense lncRNA can bind to and stabilize the 3′UTR of the Sirt1 mRNA, and gain-/ loss-of-function studies of Sirt1 antisense lncRNA demonstrate that it regulates cardiomyocyte proliferation *in vitro* and *in vivo*, and inhibition of Sirt1 antisense lncRNA suppresses cardiomyocyte proliferation (Li B. et al., 2018). Furthermore, Sirt1 antisense lncRNA overexpression promotes cardiomyocyte proliferation, improves cardiac function and decreases mortality rate after MI by interacting and stabilizing Sirt1 mRNA (Li B. et al., 2018). An lncRNA NR_045363, which is mainly expressed in cardiomyocytes and rarely non-cardiomyocytes, has been shown to stimulate cardiomyocyte proliferation and improve cardiac function in response to MI through interaction with miR-216a (Wang et al., 2019). Another recent work demonstrates that lncRNA endogenous cardiac regeneration-associated regulator (ECRAR), which is upregulated in human fetal heart, promotes DNA synthesis and cytokinesis in P7 as well as in adult rat cardiomyocytes (Chen et al., 2019). Moreover, Overexpression of ECRAR significantly stimulates cardiac regeneration and restores cardiac function after MI by targeting extracellular signal-regulated kinases 1 and 2 (ERK1/2) signaling pathway (Chen et al., 2019).

**TABLE 1:** LncRNAs in cardiac regeneration.

LncRNAs	Genomic context	Target genes	Mechanisms	Related functions	References
AZIN2-sv	Antisense	miR-214	Increase the level of PTEN and inhibit Akt/PKB signaling pathway	Repress cardiomyocyte proliferation	Li X. et al., 2018
Braveheart	Intergenic	miR-143/145	Directly interact with SUZ12 during cardiomyocyte differentiation	Maintain cardiac fate in neonatal cardiomyocytes	Klattenhoff et al., 2013
CAREL	Intergenic	miR-296	A sponge for miR-296, repress miRNA-296, following by activate Trp53inp1 and Itm2a	Repress cardiomyocyte proliferation and differentiation	Cai et al., 2018
CARMEN	Intergenic	Unknown	Interact with SUZ12 and EZH2	Promote cardiac specification and differentiation	Ounzain et al., 2015
CARL	Intergenic	miR-539	Act as an endogenous miR-539 sponge that regulates PHB2 expression, mitochondrial fission and apoptosis	Suppress Cardiomyocyte apoptosis	Wang et al., 2014
CRRL	Intergenic	miR-199a-3p	Protect Hopx from degeneration of CRRL	Suppress cardiomyocyte proliferation	Chen et al., 2018
CPR	Intergenic	DNMT3A	Interact with DNMT3A to repress the level or MCM3, promotes its methylation and inhibits its expression	Repress cardiomyocyte proliferation	Ponnusamy et al., 2019
DACH1	Intronic	PP1A	Enhance YAP1 phosphorylation and reduce ist nuclear translocation by binding PP1A	Repress cardiomyocyte proliferation	Cai et al., 2020
ECRAR	Antisense	ERK1/2	Promote the expression of cyclin D1, cyclin E1, and E2F1 proteins via ERK1/2 pathway	Promote cardiomyocyte proliferation	Chen et al., 2019
H19	Intergenic	miR-675	Regulate the expression of proliferation-associated protein 2G (PA2G4) Regulate the expression of MyoD, Myf6 and Mier2	Inhibit cardiomyocyte apoptosis Repress cardiac differentiation	Zhang et al., 2017 Ragina et al., 2012
Meg3	Intergenic	miR-145	Direct bind with RNA-binding protein FUS	Promote cardiomyocyte apoptosis	Wu et al., 2018; Chen et al., 2019
NR_045363	Intergenic	miR-216a	Promote JAK2/STAT3 signaling pathway	Promote cardiomyocyte proliferation	Wang et al., 2019
Sirt1	Antisense	Sirt1	Deacetylate and inhibit the activity of Nkx2.5, stabilized and increase the Sirt1 mRNA expression	Enhance cardiomyocyte proliferation Decrease cardiomyocyte apoptosis	Li B. et al., 2018
ST8SIA3 (RoR)	Intergenic	Unknown	Regulate the expression of genes involved in P53 response	Enhance the reprogramming of fibroblasts to cardiomyocytes	Loewer et al., 2010

In addition to lncRNAs that promote cardiac regeneration, there are also lncRNAs that suppress cardiomyocyte proliferation. The antizyme inhibitor 2 (AZIN2)-sv lncRNA is a splice variant of the AZIN2 gene that represses endogenous cardiomyocyte proliferation *in vitro* and *in vivo* (Li X. et al., 2018). In contrast, loss of AZIN2-sv promotes cellular survival and proliferation, attenuates ventricular remodeling and improves cardiac function after AMI by targeting phosphatase and tensin homology (PTEN), which blocked activation of the PI3K/Akt pathway (Li X. et al., 2018). A recent work indicates that CRRL lncRNA also suppresses cardiomyocyte proliferation by binding miR-199a-3p, while CRRL knockdown promotes neonatal rat cardiomyocyte proliferation both *in vitro* and *in vivo* (Chen et al., 2018). Loss of CRRL attenuates post-AMI remodeling and restores cardiac function in adult rats (Chen et al., 2018). Overexpression of lncRNA CAREL in mice reduces cardiomyocyte proliferation by directly binding to miR-296 (Cai et al., 2018). Conversely, loss of CAREL markedly promotes cardiac regeneration and improves cardiac function after AMI in both neonatal and adult mice (Cai et al., 2018). LncRNA cardiomyocyte proliferation regulator (CPR) overexpression remarkably suppresses neonatal cardiomyocyte proliferation and cardiac regeneration in mice (Ponnusamy et al., 2019). Conversely, CPR depletion significantly increases cardiomyocyte proliferation and restores cardiac function in postnatal and adult hearts in response to AMI in mice by directly interacting and recruiting DNMT3A to its promoter cysteine-phosphate-guanine sites (Ponnusamy et al., 2019). LncRNA dachshund homolog 1 (lncDACH1) is elevated in the postnatal hearts, and its overexpression suppresses cardiomyocyte proliferation *in vitro* and *in vivo* (Cai et al., 2020). In contrast, *in vivo* cardiac conditional knockout of lncDACH1 increases cardiomyocyte proliferation and promotes myocardial regeneration after AMI by directly binding to the protein phosphatase 1 (PP1) catalytic subunit (Cai et al., 2020). LncRNA H19 is highly conserved and downregulated in failing hearts (Viereck et al., 2020), and its inhibition promotes cardiomyocyte proliferation in P19CL6 cells during late-stage cardiac differentiation (Han et al., 2016).

Along with regulating cardiomyocyte proliferation, several lncRNAs also regulates cardiac differentiation and cardiac reprogramming. Downregulation of lncRNA H19 promotes differentiation of parthenogenetic embryonic stem cells (p-ESCs) into a higher percentage of beating cardiomyocytes (Ragina et al., 2012). Braveheart, a heart-associated lncRNA in mouse, maintains cardiac fate in neonatal cardiomyocytes by interacting with SUZ12 polycomb repressive complex 2 subunit (SUZ12), a component of polycomb-repressive complex 2 (PRC2) (Klattenhoff et al., 2013). LncRNA-ST8SIA3 (lncRNA-ROR), is a iPSC-enriched lncRNA, enhances the reprogramming of fibroblasts to cardiomyocytes (Loewer et al., 2010). Recent study shows that lncRNA CARMEN is an important regulator of cardiac differentiation, CARMEN knockdown inhibits cardiac specification and differentiation in human CPCs (Ounzain et al., 2015).

Long non-coding RNAs CARL, lncRNA maternally expressed 3 (Meg3) and lncRNA H19 are involved in cardiomyocyte apoptosis. Sirt1 antisense lncRNA not only regulates cardiomyocyte proliferation, but also regulates cardiomyocyte apoptosis. Overexpression of Sirt1 antisense lncRNA attenuates cardiomyocyte apoptosis and decrease mortality rate after AMI *in vivo* (Li B. et al., 2018). LncRNA CARL suppresses cardiomyocyte apoptosis by targeting miR-539 and PHB2 (Wang et al., 2014). LncRNA Meg3, is upregulated in the mouse heart after AMI, overexpression of Meg3 promotes cardiomyocyte apoptosis via its direct binding to RNA-binding protein FUS (Wu et al., 2018). Conversely, Meg3 deletion reduces AMI-induced cardiomyocyte apoptosis and improves cardiac function *in vivo* (Wu et al., 2018). Downregulation of lncRNA H19 inhibits cardiomyocyte apoptosis by regulating miR-19b and Sox6 in mouse P19CL6 cells (Han et al., 2016). Furthermore, knockdown of lncRNA H19 weakens cardiomyocyte apoptosis and improves myocardial function in adriamycin-induced dilated cardiomyopathy (DCM) by targeting miR-675 and Proliferation-associated protein 2G (PA2G4) (Zhang et al., 2017).

In summary, lncRNAs provide a new approach in the regulation of cardiac regeneration. However, little is known about the various and intricate regulatory mechanisms of the lncRNAs in cardiac regeneration and heart failure, which will be of particular interest in the near future.

## Part 4: Circular RNAs and Cardiac Regeneration

Circular RNA (circRNA) is a special subclass of ncRNAs characterized by a covalently closed loop structure, lacking 3′ poly(A) tails and 5′ cap structures (Mester-Tonczar et al., 2020). circRNAs are highly conserved across multiple speciesand participate in various biological processes of the cardiovascular system such as cardiomyocyte hypertrophy, cardiac regeneration and cardiac development, re, and apoptosis, which suggests that circRNAs may play a critical role in heart failure (Abbas et al., 2020). Although investigation of the role of circRNAs in cardiac regeneration has just commenced in recent years, some encouraging and promising data have already been collected, including circCDYL (Zhang et al., 2020), circ-Amot1 (Zeng et al., 2017), circFndc3b (Garikipati et al., 2019), and circNfix (Huang et al., 2019; Table 2).

**TABLE 2:** Circular RNAs in cardiac regeneration.

Circular RNAs	Mechanisms	Related functions	References
Amot1	Bind to PDK1 and AKT1	Attenuate cardiomyocyte apoptosis	Zeng et al., 2017
circCDYL	Interact with miR-4793-5p	Promote cardiomyocyte proliferation	Zhang et al., 2020
circNfix	Promote Ybx1 ubiquitin-dependent degradation and miR-214 sponge	Suppress cardiomyocyte proliferation Promote cardiomyocyte apoptosis	Huang et al., 2019
Fndc3b	Bind to FUS	Decrease cardiomyocyte apoptosis	Garikipati et al., 2019

Circular RNA circCDYL is downregulated in myocardial tissue after AMI and hypoxia myocardial cells, and also triggers cardiomyocyte proliferation. overexpression of circCDYL promotes cardiomyocyte proliferation *in vitro*, while circCDYL downregulation reduces cardiomyocyte proliferation (Zhang et al., 2020). Moreover, *in vivo* study shows that circCDYL also promotes cardiomyocytes proliferation and improves cardiac function after MI by interacting with miR-4793-5p (Zhang et al., 2020).

Circ-Amot1 is highly expressed in neonatal human cardiac tissue, overexpression of circ-Amot1 protects against doxorubicin-induced apoptosis and cardiomyopathy by binding to PDK1 and AKT1 *in vivo* (Zeng et al., 2017). Circular RNA circFndc3b is downregulated in cardiac tissues of ischemic cardiomyopathy patients, and modulates cardiac regeneration (Garikipati et al., 2019). Cardiac overexpression of circFndc3b *in vivo* attenuates cardiomyocytes apoptosis and hereby ameliorates cardiac function after MI by interacting with FUS (Garikipati et al., 2019). Circular RNA circNfix is a super enhancer-associated circRNA that is highly conserved in the hearts of rodents and humans (Huang et al., 2019). Overexpression of circNfix inhibits cardiomyocytes proliferation *in vitro* and *in vivo*, whereas circNfix downregulation promotes cardiomyocytes proliferation and decreases cardiomyocytes apoptosis after MI, attenuating cardiac dysfunction by suppressing Y-box binding protein 1 (Ybx1) ubiquitin-dependent degradation and increasing miR-214 activity (Huang et al., 2019).

To date, the field of circRNAs research is still in its infancy, particularly in the cardiac regeneration. In the future, exploration into the effects and molecular mechanisms of circRNAs on cardiac regeneration may provide new insights and therapeutic possibilities for the treatment of heart failure.

## Part 5: ncRNAs as Biomarkers and Therapeutic Targets

The adult human heart has a limited capacity to regenerate new cardiomyocytes during myocardial infarction and aging. Therefore, the endogenous cardiomyocyte renewal rate is unable to restore lost cardiomyocyte and to preserve cardiac function under physiological and pathological conditions. Benefited from the molecular and cellular discoveries as well as promising preclinical outcomes in cardiac regeneration, give us optimism in terms of regenerative therapies for the failing human heart. With the development of bioinformatics and emerging technologies, a large number of ncRNAs have been identified to regulate cardiac regeneration and are considered to be potential therapeutic targets in heart failure over the past 10 years. However, the development of ncRNAs therapy in heart failure still remains challenging.

MicroRNAs are considered ideal therapeutic targets in heart failure (Elzenaar et al., 2013). Currently, there are two general strategies to manipulate miRNAs. One is delivery of miRNA mimics or viral vector-mediated miRNA overexpression to augment the effects of miRNAs, another is to suppress the function of miRNAs by injecting anti-miRs, including antagomiRs or locked nucleic acid (LNA)-based anti-miRs (Ooi et al., 2014). AntagomiRs are 3′-cholesterol-conjugated, 2′-O-Me, 2′-fluoro, or 2′O-methoxyethyl RNA oligos of about 20–25 nucleotides, with an additional phosphorothioate backbone (Krutzfeldt et al., 2005), while LNA-based anti-miRs are antisense RNAs with several nucleotides substituted by bicyclic RNA analogs in a “locked” conformation (Lundin et al., 2013). Various studies have convincingly showed that miRNAs may improve cell therapy or enhance endogenous cardiac repair processes, and LNA-based anti-miRs have already been used in large animal models and a first clinical trial. that. For example, LNA-based anti-miR-132 treatment improves ejection fraction (EF) and ameliorates cardiac dysfunction in response to MI in pig model (Foinquinos et al., 2020). Furthermore, recent a clinical study has been launched to test the safety of CDR132L, a synthetic LNA-based antisense oligonucleotide against miR-132 (anti-miR-132), directly in heart failure patients^1^ (NCT04045405), and the results show that CDR132L is able to reverse heart failure *in vivo* (Foinquinos et al., 2020). In addition, study shows that anti-miR-92a increases cardiac regeneration, reduces infarct size and protects against Ischemia-reperfusion (I/R) injury in pig model (Hinkel et al., 2013). MRG-110 is a LNA-based antisense oligonucleotide that targets miR-92a. Recently, MRG-110 has been tested in healthy human adults and MRG-110 treatment reduces miR-92a level and de-represses the target genes in human peripheral blood cells (European Clinical Trials Database [EudraCT] No. 2017-004180-12) (Abplanalp et al., 2020). Currently, the miRNA-based LNAs treatment is in clinical trials and receives positive feedback on their potentials, but none of them have been reached in the pharmaceutical breakthrough yet. Therefore, the development of miRNA therapeutic tools is still a long process, and a multitude of companies will put more efforts on the miRNA therapeutics in the next several years to bring them from the bench to the market.

The potential for identifying novel lncRNAs involved in the regulation of cardiac regeneration is fairly high. Unfortunately, the identification of novel lncRNAs is more limited due to the lack of libraries to perform systematic screenings, since all the current lncRNAs regulating cardiac regeneration were identified in the last very few years. It has been shown that lncRNAs can be detected in the plasma or urine and display a dynamic alteration upon initiation and progression of heart failure. Recent studies show that LncRNA long intergenic non-coding RNA predicting cardiac remodeling (LIPCAR), smooth muscle and endothelial cell-enriched migration/differentiation-associated long non-coding RNA (SENCR) and myocardial infarction associated transcript (MIAT) can be detected in the plasma and serve as potential markers of heart failure (Hobuss et al., 2019). To date, the development of lncRNA therapy in heart failure is still in its infancy. However, lncRNAs potentially offer a promising new therapeutic approach to treat heart failure in the future. Therefore, to study the molecular mechanisms of lncRNAs and to identify novel lncRNAs in cardiomyocytes will provide a new insight into understanding of the lncRNAs in heart failure.

The number of ncRNAs implicated in the regulation of cardiac regeneration is certainly expectable to increase in the near future, making ncRNAs-based therapy is a very promising approach for the treatment of heart failure. However, an in-depth and thorough understanding of ncRNAs molecular targets and regulatory mechanisms are necessary to develop more efficient, efficacious, and safe therapeutic strategies for treatment of heart failure patients. Thus, there is an urgent need for further development of ncRNAs for the therapy of Heart failure in both animal models and human clinic trials. One miRNA/lncRNA can bind up to several hundreds of mRNAs thereby affecting gene expression networks. In addition, one mRNA can be targeted by several miRNAs/lncRNAs highlighting the complex network of interactions. Therefore, a better understanding the detailed functions and molecular mechanisms of ncRNAs in cardiac regeneration need to be further explored. Additionally, repairing the failing human heart may necessitate a combination of multiple therapeutic approaches. Additionally, repairing the human failing heart may necessitate the use of combinatorial therapeutic approaches to overcome the many innate hurdles of cardiac regeneration. Thus, a combinatorial ncRNA targeting strategy needs to be considered in the path to novel MI therapeutics development.

## Author Contributions

TY wrote the manuscript and put the Figure and tables together. JK edited the manuscript, the figure and tables, and helped to structure and revise the manuscript. Both authors contributed to the article and approved the submitted version.

## Conflict of Interest

The authors declare that the research was conducted in the absence of any commercial or financial relationships that could be construed as a potential conflict of interest.

## Footnotes

1 https://clinicaltrials.gov
